# SAHA attenuates Takotsubo-like myocardial injury by targeting an epigenetic Ac/Dc axis

**DOI:** 10.1038/s41392-021-00546-y

**Published:** 2021-04-20

**Authors:** Ishant Khurana, Scott Maxwell, Simon Royce, Prabhu Mathiyalagan, Tom Karagiannis, Nadia Mazarakis, Jitraporn Vongsvivut, Harikrishnan K.N., Jun Okabe, Keith Al-Hasani, Chrishan Samuel, Assam El-Osta

**Affiliations:** 1 Human Epigenetics Program, Department of Diabetes, Central Clinical School, Monash University, Melbourne, VIC Australia; 2 Cardiovascular Disease Program, Monash Biomedicine Discovery Institute and Department of Pharmacology, Monash University, Clayton, VIC Australia; 3 Infrared Microspectroscopy Beamline, ANSTO - Australian Synchrotron, Clayton, VIC Australia; 4 Department of Medicine and Therapeutics, The Chinese University of Hong Kong (CUHK), Hong Kong SAR; Hong Kong Institute of Diabetes and Obesity, CUHK, Hong Kong SAR; Prince of Wales Hospital, Li Ka Shing Institute of Health Sciences, CUHK, Shatin, Hong Kong, China; 5 University College Copenhagen, Faculty of Health, Department of Technology, Biomedical Laboratory Science, Copenhagen, Denmark

**Keywords:** Cardiology, Epigenetics

**Dear Editor**,

Takotsubo syndrome (TS) is a stress-induced non-ischaemic cardiomyopathy that is more common in women, but is associated with higher morbidity and mortality in males. Also known as broken-heart syndrome, TS is characterised by transient left ventricular (LV) dysfunction independent of obstructive coronary artery disease. TS is a polygenic condition and nowhere is this more evident than the use of positive inotropes, such as isoprenaline (ISO) in pre-clinical models.^1^ There is no standard therapy for broken-heart syndrome because the mechanisms underlying the condition remain unknown. Furthermore, there is no consensus on predisposition for Takotsubo^2^ and our goal was to better understand the regulatory mechanism as a first step towards improved treatment plans. Suberanilohydroxamic acid or SAHA, a drug approved for cancer treatment by the US Food and Drug Administration has previously been shown to improve cardiopulmonary function.^3^ We tested the hypothesis that the cardioprotective benefit of SAHA in a pre-clinical model of Takotsubo is conferred by an epigenetic acetylation/deacetylation (Ac/Dc) axis.

Eight-week-old 129/Sv mice were divided into four groups of five animals. Group 1 received intraperitoneal (IP) saline and served as healthy controls. Mice in group 2 served as a SAHA only control receiving IP injections every third day (100 mg/kg, illustrated by red arrows). The remaining groups (3 and 4) received subcutaneous isoprenaline (ISO, 25 mg/kg) once daily for the first 5 days to induce cardiomyopathy (illustrated by blue arrows). The remaining ISO-injury mice were therapeutically administered SAHA (ISO/SAHA). Group 4 commenced SAHA treatment on the day after ISO administration was concluded (ISO/SAHA, referred to as Reversal or “REV”) (Fig. 1a).

**Fig. 1:**
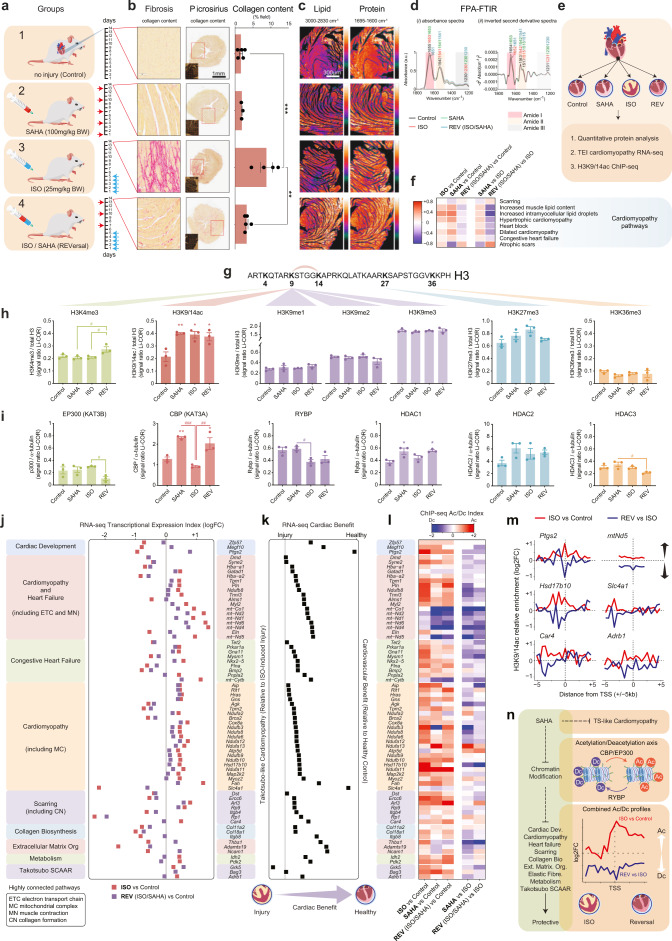
Therapeutic benefit of SAHA targeting the Ac/Dc axis. **a** Illustration of ISO-induced Takotsubo-like cardiomyopathy. In the pre-clinical model, mice were divided into four groups. Group 1 received IP saline and served as healthy controls. Group 2 served as a SAHA only control receiving IP injections every third day. The remaining groups (3 and 4) received subcutaneous ISO once daily for the first 5 days to induce TS-like cardiomyopathy. Group 4 commenced SAHA treatment on the day after ISO administration was concluded (REVersal). Mice were sacrificed 9 days following administration of the fifth ISO dose, on day 14. **b** Quantitative assessment of LV collagen content. Representative picrosirius red-stained images are shown. Data presented as the mean ± SEM. Control vs. ISO, exact *P*-value 0.0001 (***), ISO vs. REVersal, exact *P*-value 0.001 (**). Calculated using unpaired *t*-test. Picrosirius red staining also shown. Control (*n* = 5), SAHA (*n* = 3), ISO (*n* = 5) and Reversal (ISO/SAHA *n* = 5). **c** FPA-FTIR chemical images showing lipid and protein distribution using a Bruker Hyperion 2000 FTIR microscope equipped with a 64 × 64 element FPA detector and a 15× objective lens and acquired in transmission mode. **d** Comparison of FPA-FTIR (*i*) absorbance spectra and (*ii*) inverted second derivative spectra within the amide region (1800–1200 cm^−1^). **e** Apical LV tissues were assessed for TS-like response to SAHA treatment using Li-COR protein quantification, transcriptional expression index (TEI) determined by RNA-seq and chromatin modification by ChIP-seq. **f** Heatmap of cardiomyopathy pathways from multi-contrast GSEA derived from the Human Phenotype Ontology (HPO). Pathway significance is calculated by FDR *P* < 0.05 using multi-contrast analysis for ISO vs. healthy controls, SAHA vs. healthy controls, ISO/SAHA vs. healthy controls (REV), SAHA vs. ISO and ISO/SAHA vs. ISO (REV) (*n* = 3 each group). **g** Histone lysine map for the major sites assessed of H3 acetylation and methylation, these include the transcriptionally permissive marks H3K4me3, H3K9/14ac, and H3K36me3. The suppressive marks analysed include H3K9me1, H3K9me2, H3K9me3, H3K27me3. Lysine acetylation at positions K9 and K14 are linked in the schematic. **h** Protein levels of histone modification in apical LV tissue from ISO and SAHA-administered mice. Bar plots represent mean values of Li-COR Odyssey quantification of protein adjusted against total histone H3. Data are represented as the mean ± SEM. *P < 0.05, ** *P* < 0.01 vs. control, #*P* < 0.05. Calculated using one-way ANOVA (*n* = 3 each group). **i** Protein levels of histone-modifying enzymes (lysine acetyltransferases EP300 and CBP including the deacetyltransferases RYBP, HDAC1, HDAC2 and HDAC3) in apical LV tissue from ISO and SAHA-administered mice. Bar plots represent mean values of Li-COR Odyssey quantification of protein adjusted against total α-tubulin. Data are represented as the mean ± SEM. *P < 0.05, **P < 0.01 vs. control, #*P* < 0.05, ##*P* < 0.01, ###*P* < 0.001. Calculated using one-way ANOVA (*n* = 3 each group). **j** RNA-seq data derived from apical LV shows the transcriptional expression index (TEI) of genes in the major cardiomyopathy pathways identified above (**f**) in ISO-induced injury and influenced by SAHA treatment. Pathways include cardiac development, cardiomyopathy and heart failure, congestive heart failure, cardiomyopathy, cardiac scarring, collagen biosynthesis, extracellular matrix organisation, cardiac metabolism and genes associated with genetic variation for Takotsubo derived from SCAAR (Swedish Coronary Angiography and Angioplasty Registry). Highly connected pathways include electron transport chain (ETC), mitochondrial complex (MC), muscle contraction (MN) and collagen formation (CN). Gene–gene name (filtered by FDR *P*-value < 0.05, annotated to mm10 mouse genome); transcription expression index–median gene expression of transcripts in REV (*n* = 3, ISO/ SAHA) and ISO (*n* = 3) groups relative to healthy controls (*n* = 3) expressed as log2 fold-change. **k** Cardiac benefit of SAHA treatment in ISO-induced model of Takotsubo. Transcriptional expression index is shown for genes influenced by SAHA in the reversal model of ISO-induced cardiomyopathy. Genes implicated with Takotsubo-like injury are shown on the left of the axis (control relative to ISO-induced injury groups). Transcribed genes that shift to the right are closer to healthy control in the SAHA treatment group; REVersal (control vs. ISO/SAHA groups) *n* = 3/treatment group. **l** Heatmap of H3K9/14ac ChIP-seq signals relative to the TSS ( + /− 2500 bp) of genes identified above in panel **j**. Left insert columns show log2(fold-change) of H3K9/14ac signals for ISO vs. healthy controls, SAHA vs. healthy controls and (REV) ISO/SAHA vs. healthy controls. Elevated H3K9/14 acetylation (Ac) signals are shown in red on the colour gradient and the reduction of H3K9/14 acetylation or deacetylation (Dc) signals are shown in blue. No change in H3K9/14ac signal is denoted as white and grey is undetermined. Right insert columns show H3K9/14ac signals for SAHA vs. ISO and (REV) ISO/SAHA vs. ISO groups. Control (*n* = 5), ISO (*n* = 5), SAHA (*n* = 3) and REV (*n* = 5). **m** ChIP-seq contour profiles for H3K9/14ac relative to the TSS+/−5kb are shown for several representative genes and their associated pathways; *Ptgs2* (cardiac development)*, mtNd5* (cardiomyopathy and heart failure)*, Hsd17b10* and *Slc4a1* (cardiomyopathy)*, Car4* (cardiac scarring) and *Adrb1* (Takotsubo SCAAR). Control (*n* = 5), ISO (*n* = 5), SAHA (*n* = 3) and REV (*n* = 5). **n** Proposed cardiac benefit of SAHA treatment in TS-like cardiomyopathy involves an Ac/Dc axis implicating the CBP/EP300 acetyltransferases and RYBP deacetyltransferase. SAHA treatment influences core genes associated with the Ac/Dc axis and cardiomyopathy. Combined ChIP-seq Ac/Dc profiles are shown for cardiomyopathy genes identified above in panels **j**–**l** (red Ac profile represents ISO vs. Control and blue Dc profile represents REV vs. ISO). Control (*n* = 5), ISO (*n* = 5), SAHA (*n* = 3) and REV (*n* = 5)

ISO significantly increased picrosirius-stained collagen content in the infarcted LV (Fig. 1b, group 3). SAHA treatment attenuated this ISO-induced collagen content (Fig. 1b, group 4). As protein accumulation is characteristic of TS, we assessed collagen deposition.^4^ SAHA treatment attenuated ISO-induced injury by decreasing collagen. Quantitative histological analysis confirmed that LV collagen content was ~3% for untreated and SAHA only groups when compared to 8% in the ISO-induced injury group. Collagen deposition in the REV group was reduced to 4%. These findings indicated that treatment with SAHA reduced ISO-induced LV collagen deposition.

Since the area of ISO-induced injury was vastly improved following SAHA treatment, we assessed protein accumulation using chemical mapping. For each tissue section, an FPA-FTIR image of 6 × 8 grids were collected on the area of infarction of 1.04 × 1.38 mm^2^ (Fig. 1c). The absorbance spectra showed a decrease in the intensity of the amide I band (α-helix secondary protein structure) and that was correlated with an increase in the amide II band (Fig. 1d, i). The amide I/II intensity ratios obtained from the inverted second derivative spectra confirmed these changes, and showed a decrease in all treatment groups (ISO = 1.8, SAHA = 1.8, ISO/SAHA = 1.6) when compared to the control group (2.0) (Fig. 1d, ii). The SAHA group had an increased intensity at 1515 cm^−1^ that was associated with α-helical protein structures. Given that collagen is largely comprised of α-helical structures, the FPA-FTIR imaging results were consistent with histological observations. Taken together, this data suggested that treatment with SAHA influenced the composition of secondary protein structures in ISO-induced injury.

The transcriptome of Takotsubo and how SAHA influences gene behaviour is poorly understood. Apical LV tissues were assessed for Takotsubo-like response to SAHA treatment using Li-COR protein quantification, transcriptional expression index (TEI) by RNA-seq (sequencing) and H3K9/14 acetylation/deacetylation by ChIP-seq (Fig. 1e). Gene set enrichment analysis (GSEA) identified pathways implicated in ISO-induced cardiomyopathy were vastly improved by SAHA (Fig. 1f). As these results suggested that treatment with the hydroxamic acid could influence cardiac remodelling, we assessed actively transcribed genes that belong to these highly connected pathways. Histone H3 acetylation (ac) and methylation (me) are powerful determinants that regulate cardiac transcription. We assessed H3K9/14ac, H3K4me3, H3K9me1, H3K9me2, H3K9me3, H3K27me3 and H3K36me3 (Fig. 1g). Li-COR analyses showed a dramatic change in H3K9/14ac following ISO-induced injury and SAHA treatment (Fig. 1h). H3K4me3 another permissive mark of gene transcription was also elevated in SAHA-treated animals (REV). Since the acetyltransferases CBP (KAT3A) and EP300 (KAT3B) are known to regulate cardiac transcription and are subject to the Ac/Dc axis by SAHA,^5^ we assessed their protein levels. We also examined co-repressive deacetyltransferases RYBP, HDAC1, HDAC2 and HDAC3. We showed that CBP (KAT3A) protein levels were reduced in ISO-induced cardiomyopathy but reversed by SAHA treatment when compared to control animals (no injury) (Fig. 1i). Histone acetyltransferase EP300 (KAT3B) was significantly reduced in the REV group with RYBP reduced in ISO-induced cardiomyopathy animals. These results suggested the Ac/Dc modifiers that influence ISO-induced cardiomyopathy were attenuated by SAHA.

Pathways directly regulated by ISO-induced cardiomyopathy and reversed by SAHA included cardiac development, heart failure, congestive heart failure, cardiomyopathy, cardiac scarring, collagen biosynthesis, extracellular matrix organisation and cardiac metabolism (Fig. 1j). We also assessed genetic candidates associated with predisposition to Takotsubo derived recently from the Swedish Coronary Angiography and Angioplasty Registry (SCAAR).^2^ RNA-seq shows SAHA treatment influenced the TEI (transcriptional expression index) of genes with a shift towards cardiac benefit (Fig. 1k). These results suggested that the core pathways identified could be regulated by SAHA treatment. The recent SCAAR screen found no genetic link with Takotsubo for polymorphisms in the *Adrb1*, *Bag3* and *Grk5* genes.^2^ However, we observed a dramatic shift in the TEI following SAHA treatment, suggesting the expression of stress-induced genes could also be under epigenetic control.

While total histone acetylation is often assessed by protein blotting it is not informative of genomic location. To determine whether the cardiac benefit conferred by SAHA treatment was regulated by an Ac/Dc axis, we performed chromatin immunoprecipitation assays from LV tissues followed by sequencing (ChIP-seq). We assessed H3K9/14ac signals in all groups. SAHA treatment influenced acetylation and deacetylation of genes implicated with Takotsubo (Fig. 1l). Surprisingly, this included prominent lysine deacetylation of the SCAAR-associated genes *Adrb1*, *Bag3* and *Grk5*.^2^ These results highlighted that the myocardial transcriptome was not only influenced by ISO—but for the core genes identified in this study— lysine acetylation at transcribed genes were either partially or fully restored by SAHA (Fig. 1m).

This is the first detailed description of the cardiac transcriptome using SAHA as a therapeutic drug in a model of Takotsubo. We propose that the expression of genes that are influenced by SAHA are neither random nor limited to any one gene, but rather, co-ordinate pathways that preserve cardiac function. While H3K9/14ac selectivity was previously described for EP300 gene targets^5^ in this study we observed the expression of stress-induced genes were influenced in the REV model (ISO/SAHA vs. Control) as well as the Ac/Dc index in the REV (ISO/SAHA) vs. ISO groups. Further work is required to learn the regulatory components such as CBP/EP300 and other RYBP-associated HDAC components because of the paradoxical Ac/Dc nexus^5^ (Fig. 1n). In conclusion, the expanded gene list described for Takotsubo-like cardiomyopathy is an important step towards establishing an epigenetic framework to further understand the effects of HDAC inhibitors and the continued development of compounds like SAHA that have cardioprotective benefits for broken-heart syndrome.

## Supplementary information

Supplemental Material

## Data Availability

The datasets analysed in the current study are available from the corresponding author on reasonable request.

## Study approval

This study was approved by the Monash University Animal Ethics Committee.
