# Supplementary material for: SAHA attenuates Takotsubo-like myocardial injury by targeting an epigenetic Ac/Dc axis
Source: Signal Transduct Target Ther. 2021 Apr 20;6:159. doi: 10.1038/s41392-021-00546-y (PMC8055670; doi:10.1038/s41392-021-00546-y)
Supplement: Supplementary file 1 — Supplemental Material [file 41392_2021_546_MOESM1_ESM.docx]

Supplementary Materials for

SAHA Attenuates Takotsubo-like Myocardial Injury by Targeting an Epigenetic Ac/Dc Axis

Running title; Targeting the Ac/Dc Axis in Broken Heart Syndrome

Ishant Khurana, Scott Maxwell, Simon Royce, Prabhu Mathiyalagan, Tom Karagiannis, Nadia Mazarakis, Jitraporn Vongsvivut, Harikrishnan KN, Jun Okabe, Keith Al-Hasani, Chrishan Samuel and Assam El-Osta

Correspondence to: [sam.el-osta@monash.edu](mailto:sam.el-osta@monash.edu)

**This PDF file includes:**

Materials and Methods

**Materials and Methods**

**ISO-induced** **cardiomyopathy**

Twenty, eight-week-old 129/Sv mice were given ad libitum access to food and water (12-hour light-dark cycle) and maintained according to the Australian guidelines for the care and use of laboratory animals for scientific purposes (study approved by the Monash University animal ethics committee). Animals were divided into four groups. One group of mice received intraperitoneal (IP) saline and served as a control. Two groups received subcutaneous isoproterenol (ISO, 25 mg/kg) once daily for the first five days of the study to cause ischemia-induced cardiomyopathy. Subgroups of ISO-treated mice were administered with SAHA (IP, 100 mg/kg) every third day. SAHA treatment commenced on the day after ISO treatment was concluded (post-treatment; reversal). The final group of mice served as a SAHA only control (IP, 100 mg/kg). Mice were sacrificed nine days following administration of the fifth dose of ISO (total 14 days). Following the study, formalin-fixed, paraffin-embedded left ventricle were sectioned (4 µm in thickness) onto either histological or calcium fluoride (CaF_2_) windows, and de-paraffinised by two consecutive washes of xylene.

**FPA-FTIR chemical imaging**

The thin sections of formalin-fixed, paraffin-embedded left ventricle mounted on the CaF_2_ windows were used for focal plane array-Fourier transform infrared (FPA-FTIR) microspectroscopic measurement at ANSTO - Australian Synchrotron (Clayton, Victoria, Australia). FPA-FTIR chemical images showing lipid and protein distribution were acquired using a Bruker Hyperion 2000 FTIR microscope equipped with a liquid nitrogen cooled 64 × 64 element FPA detector and a 15× objective lens, coupled to a Vertex 70 FTIR spectrometer (Bruker Optik GmbH, Ettlingen, Germany). The data acquisition was performed in transmission mode within a 4000–800 cm^−1^ spectral region, in forms of 6 × 8 grids covering a total sampling area of 1.04 × 1.38 mm^2^. For each tissue sample, high-quality FPA-FTIR spectral images were collected at 8-cm^−1^ resolution, with 64 co-added scans, Blackman-Harris 3-Term apodization, Power-Spectrum phase correction, and a zero-filling factor of 2 using OPUS 7.2 imaging software (Bruker). Background measurements were taken prior to sample spectral images, by focusing on a clean surface area of the CaF_2_ substrate using the same acquisition parameters. The areas of interest on the samples were selected based on their corresponding picrosirius red stained images. Spectra were first subjected to an atmospheric water correction and vector normalization prior to performing area integration of lipid bands (CH_2_ stretching modes: 3000–2830 cm^-1^) and protein band (amide I: 1695–1600 cm^-1^). Comparison of average FPA-FTIR spectra extracted from the area of myocardial infarction included the SAHA-treated and control groups, ISO-induced injury and the control groups, and the reversal of SAHA treatment with the control groups.

**Li-COR Odyssey protein quantification**

Protein levels of histone modification in LV from ISO and SAHA administered mice using Li-COR Odyssey quantification of protein adjusted against total histone H3 as previously described^1^. Histone map for the major sites assessed of H3 acetylation and methylation used antibodies that specifically recognise H3K4me3, H3K9/14ac, H3K9me1, H3K9me2, H3K9me3, H3K27me3 and H3K36me3. Protein levels of histone modifying enzymes (lysine acetyltransferases EP300 (KAT3B) and CBP (KAT3A) including the deacetyltransferases RYBP, HDAC1, HDAC2 and HDAC3) in LV from ISO and SAHA administered mice. Bar plots represent mean values of Li-COR Odyssey quantification of protein adjusted against total α-tubulin.

**RNA library preparation and sequencing**

We systematically examined RNA transcripts by deep sequencing using protocols previously described by the group ^2^. Samples were homogenized or lysed in TRIzol^®^ (Life Technologies, Waltham, MA) and RNA was isolated using the Direct-zol™ RNA Mini prep kit (Zymo Research, Irvine, CA) according to the manufacturer’s instructions. cDNA synthesis and qRT-PCR analysis of gene expression were performed as previously described^3,4^. NEBNext^®^ Poly(A) mRNA Magnetic Isolation Module (New England Biolabs, Ipswich, MA) was used to enrich mRNA from 1μg of total RNA. Barcoded libraries were generated using the NEBNext^®^ Ultra™ Directional RNA Library Prep Kit for Illumina^®^ (New England Biolabs) following the manufacturer’s instructions. Deep sequencing was performed using Illumina Hiseq 2500 (San Diego, CA) using version 4 kits for 100 cycles at the Australian Genome Research Facility (Melbourne, Australia). Sequence reads underwent quality trimming with Fastx-Toolkit (<http://hannonlab.cshl.edu/fastx_toolkit/>). Trimmed reads were mapped to the mouse genome using STAR aligner^5^. Tags aligning to genes were counted using FeatureCounts with Ensembl annotations. Changes in gene expression were determined using edgeR^6^. Genes with less than 10 reads average across all samples were excluded from the analysis, and the false discovery rate (FDR) threshold was set equal to or less than 0.05.

**Rank-rank and gene set enrichment analysis**

Gene Set Enrichment Analysis (GSEA)^7^ was conducted on differentially expressed genes ranked according to this R calculation: -log10(‘p-value’) * sign(‘log-fold-change’), with examination of gene sets of the Reactome pathways database v7.

**Chromatin immunoprecipitation**

Chromatin immunoprecipitation assays were performed previously described^8,9^. LV tissues were fixed for 10 minutes with 1% formaldehyde. Glycine (0.125 M) solution was then added for another 10 minutes. Fixed left ventricle tissues were resuspended in sodium dodecyl (lauryl) sulfate (SDS) lysis buffer (1% SDS, 10 mM EDTA, 50 mM Tris-HCl pH 8.1) including a protease inhibitor cocktail (Roche Diagnostics GmBH, Mannheim, Germany) and homogenized to cell suspension followed by incubation on ice for 5 minutes. Solubilised samples were sonicated to shear chromatin to 200-600 bp. Chromatin was resuspended in ChIP Dilution Buffer (0.01% SDS, 1.1% Triton X-100, 1.2 mM EDTA, 16.7 mM Tris-HCl pH 8.0 and 167 mM NaCl) and 20 µl of Dynabeads® Protein A (Invitrogen, Carlsbad, CA, USA) was added and pre-cleared. H3K9/14ac antibody (06-599, Millipore, Burlington, MA, USA) was used for immunoprecipitation of soluble chromatin and incubated overnight at 4˚C. Immunoprecipitated complexes were collected by magnetic isolation, washed low salt followed by high salt buffers and eluted with 0.1 M NaHCO_3_ with 1% SDS. Protein-DNA cross-links were reversed by adding Proteinase K (Sigma, St. Louis, MO, USA) and incubation at 62˚C for 2 hours. DNA was recovered using a Qiagen MinElute column (Qiagen Inc., Valencia, CA, USA).

**H3K9/14ac library preparation and sequencing**

ChIP-seq libraries were prepared using Illumina protocols (San Diego, CA, US) as previously described^8,9^. Briefly, 10 ng of immunopurified DNA or genomic DNA from an input sample was end repaired, followed by 3’ adenylation using Klenow fragment (3’ to 5’ exo minus) and ligated to universal library adapters with T- overhangs at their 3’ end. Ligated material was separated on a 2.0% agarose gel, and then fragments in the range of 250-350 bp were excised and column purified (Qiagen). A DNA library was obtained by an 18 cycle PCR amplification (10s 98˚C, 30s 65˚C, 30s 72˚C) using Phusion DNA polymerase (Invitrogen) with oligonucleotides complementary to Illumina sequencing adapters. Column purified libraries were quantified using Qubit fluorometer (Life Technologies) and visualised by MultiNA capillary electrophoresis and DNA-500 kit (Shimadzu, Kyoto, Japan) for quality assurance. Libraries were diluted to 10 nM and stored at -20°C prior to cluster generation. Short reads were aligned with BWA using default parameters^10^. Regions of interest were defined using MACS software using default parameters^11^. For ChIP-seq overlapping regions of histone modification in the SAHA treated and untreated animals were merged to create a single list of regions as determined by MACS. H3K9/14ac profiles were determined by CoverageView using 0.5 Kb regions on the TSS (+/-5Kb)^12^. Data from SAHA treated animals was analysed using MACS peak calling by comparing each sample to the input, using default settings.

**References**

1 Kaipananickal, H. *et al.* Targeting Treatment Refractory NET by EZH2 Inhibition in Postural Tachycardia Syndrome. *Circ Res* **126**, 1058-1060, (2020).

2 Quaife-Ryan, G. A. *et al.* Multicellular Transcriptional Analysis of Mammalian Heart Regeneration. *Circulation* **136**, 1123-1139, (2017).

3 Okabe, J. *et al.* Distinguishing hyperglycemic changes by Set7 in vascular endothelial cells. *Circ Res* **110**, 1067-1076, (2012).

4 Tuano, N. K. *et al.* Set7 mediated interactions regulate transcriptional networks in embryonic stem cells. *Nucleic Acids Res* **44**, 9206-9217, (2016).

5 Dobin, A. *et al.* STAR: ultrafast universal RNA-seq aligner. *Bioinformatics* **29**, 15-21, (2013).

6 Robinson, M. D., McCarthy, D. J. & Smyth, G. K. edgeR: a Bioconductor package for differential expression analysis of digital gene expression data. *Bioinformatics* **26**, 139-140, (2010).

7 Subramanian, A. *et al.* Gene set enrichment analysis: a knowledge-based approach for interpreting genome-wide expression profiles. *Proc Natl Acad Sci U S A* **102**, 15545-15550, (2005).

8 Rafehi, H. *et al.* Vascular histone deacetylation by pharmacological HDAC inhibition. *Genome Res* **24**, 1271-1284, (2014).

9 Pirola, L. *et al.* Genome-wide analysis distinguishes hyperglycemia regulated epigenetic signatures of primary vascular cells. *Genome Res* **21**, 1601-1615, (2011).

10 Li, H. & Durbin, R. Fast and accurate long-read alignment with Burrows-Wheeler transform. *Bioinformatics* **26**, 589-595, (2010).

11 Zhang, Y. *et al.* Model-based analysis of ChIP-Seq (MACS). *Genome Biol* **9**, R137, (2008).

12. Lowy E. CoverageView: Coverage visualization package for R. *R package version 1.28.0.* doi:[10.18129/B9.bioc.CoverageView](https://doi.org/doi:10.18129/B9.bioc.CoverageView" \o "DOI for use in publications, etc., will always redirect to current release version (or devel if package is not in release yet).) (2020).
